# The Genome Sequences of Baculoviruses from the Tufted Apple Bud Moth, *Platynota idaeusalis*, Reveal Recombination Between an Alphabaculovirus and a Betabaculovirus from the Same Host

**DOI:** 10.3390/v17020202

**Published:** 2025-01-30

**Authors:** Robert L. Harrison, Michael A. Jansen, Austin N. Fife, Daniel L. Rowley

**Affiliations:** 1 Invasive Insect Biocontrol and Behavior Laboratory, Beltsville Agricultural Research Center, USDA Agricultural Research Service, Beltsville, MD 20705, USA; daniel.rowley@usda.gov; 2 Systematic Entomology Laboratory, Beltsville Agricultural Research Center, USDA Agricultural Research Service, Beltsville, MD 20705, USA; andrew.jansen@usda.gov (M.A.J.); austin.fife@usda.gov (A.N.F.)

**Keywords:** baculovirus, *Alphabaculovirus*, *Betabaculovirus*, *Platynota idaeusalis*, nucleopolyhedrovirus, granulovirus, genome, recombination, occlusion bodies

## Abstract

The USDA-ARS collection of insect viruses at Beltsville, MD, USA, contains samples of an alphabaculovirus from larvae of the tufted apple bud moth, *Platynota idaeusalis* Walker, as well as a presumptive betabaculovirus from the same host species. The viruses in these samples—Platynota idaeusalis nucleopolyhedrovirus isolate 2680 (PlidNPV-2680) and Platynota idaeusalis granulovirus isolate 2683 (PlidGV-2683)—were characterized by electron microscopy of their occlusion bodies (OBs) and determination and analysis of their genome sequences. Scanning and transmission electron microscopy of the OBs revealed morphologies typical for alphabaculoviruses and betabaculoviruses. Sequencing viral DNA resulted in circular genomes of 121,881 bp and 106,633 bp for PlidNPV-2680 and PlidGV-2683, respectively. Similar numbers of ORFs (128 for PlidNPV-2680, 125 for PlidGV-2683) were annotated, along with ten homologous regions (*hr*s) in the PlidNPV-2680 genome and five intergenic regions of tandem direct repeats (*dr*s) in the PlidGV genome. Phylogenetic inference from core gene alignments suggested that PlidMNPV-2680 represents a unique lineage within the genus *Alphabaculovirus*, while PlidGV-2683 was grouped with clade b betabaculoviruses. A comparison of the PlidNPV-2680 and PlidGV-2683 genomes revealed a 1516 bp region in PlidNPV-2680 that exhibited 97.5% sequence identity to a region of the PlidGV-2683 genome, suggesting that recombination had occurred recently between viruses from these lineages.

## 1. Introduction

Baculoviruses are large dsDNA viruses of insects that are known for their distinctive virion-containing occlusion bodies (OBs) produced during infection and for their development as biopesticides and as gene expression vectors with many applications [1,2,3,4]. These viruses are classified in the family *Baculoviridae*, which, in turn, is a member family of the order *Lefavirales,* along with the families *Nudiviridae* and *Hytrosaviridae* [5,6]. *Lefavirales* is further classified in the order *Naldaviricetes* with the family *Nimaviridae* [6]. Naldaviricetes are characterized by having arthropod hosts, producing rod-shaped virions that contain large circular dsDNA genomes, and encoding homologs of envelope proteins known as *per os* infectivity factors (*pif*s). In baculoviruses, *pif* proteins play an essential role in the primary infection of midgut cells of host larvae that ingest occlusion bodies [7,8]. Lefavirals are naldaviricetes that contain homologs of late expression factors (*lef*s), parts of the baculovirus late-phase transcriptional complex, including the four subunits of the baculovirus RNA polymerase [9].

Baculovirus genomes range in size from approximately 80 to 180 kbp and contain about 90 to 180 annotated open reading frames (ORFs). A core set of 38 conserved genes has been identified in all baculovirus genomes [10,11]. The development of high-throughput DNA sequencing technologies has allowed for the complete genome sequence determination of many baculoviruses, almost all of which are from the genera *Alphabaculovirus* (nucleopolyhedroviruses; NPVs) and *Betabaculovirus* (granuloviruses; GVs) [5]. These viruses infect larvae of moths and butterflies (order Lepidoptera) and usually cause a dramatic pathology characterized by the lysis of cells and tissues, the liquefaction of the host’s internal anatomy, and post-mortem rupture of the host cuticle accompanied by the environmental release of progeny OBs [1,12,13]. Epizootics caused by these viruses and the striking nature of baculovirus disease have led to the identification and collection of many baculoviruses from the field. Genome sequences from field isolates have provided information on the diversification of, and variation among, viruses of this family.

In 1974, a baculovirus was identified in diseased larvae of the tufted apple bud moth (TABM), *Platynota idaeusalis* (Walker), in Pennsylvania, USA [14]. TABM is a moth of the family Tortricidae that has been identified throughout the eastern half of North America and the Pacific Northwest [15]. Although TABM larvae have been found feeding on a diverse range of host plants, TABM has drawn attention as a significant direct pest of apples in the mid-Atlantic USA [16]. The baculovirus that was discovered in TABM larvae also exhibited virulence towards two other *Platynota* species in addition to *P. idaeusalis*. Initial molecular and ultrastructural characterization of the viral DNA and OBs indicated that this virus was an alphabaculovirus [14,17].

A sample of this virus was sent to scientists at the USDA-ARS laboratory in Beltsville in 1975. In addition, the isolates of a second, undescribed virus from TABM, labeled as a granulovirus (genus *Betabaculovirus*), were deposited in the Beltsville insect virus collection in 1981. To further identify and characterize these viruses, we carried out electron microscopy on their occlusion bodies and sequenced and analyzed the genome sequences of the alphabaculovirus isolate (Platynota idaeusalis nucleopolyhedrovirus 2680; PlidNPV-2680) and one of the betabaculovirus isolates (Platynota idaeusalis granulovirus 2683, PlidGV-2683). Our studies revealed that both viruses are previously unclassified members of the family *Baculoviridae*. Analysis of the genome sequences revealed an instance of recent recombination between viruses from the lineages represented by PlidNPV-2680 and PlidGV-2683.

## 2. Materials and Methods

### 2.1. Virus Samples

A sample of NPV occlusion bodies extracted from *P. idaeusalis* larvae was obtained from W. M. Bode at Pennsylvania State University in May 1975. The virus was designated isolate 2680 and deposited in the USDA-ARS insect virus collection in Beltsville, MD (https://www.gbif.org/grscicoll/collection/9f84b54d-2829-4934-afc2-e49130e1bc14) (accessed 27 September 2023).

Occlusion body samples of three GV isolates from TABM larvae from an unlisted source were deposited in the Beltsville virus collection on 2 April 1981. Isolate 2683 was selected for further study.

Both virus samples consisted of extracted OBs in aqueous suspension.

### 2.2. Electron Microscopy

#### 2.2.1. LT-SEM

Both samples of baculovirus OBs were imaged using a Hitachi SU7000 Schottky-Emitter SEM. The SEM was outfitted with a Quorum-brand sample preparation chamber, a nitrogen slushing pot, and a liquid nitrogen pumping system. Both the preparatory chamber and SEM stage were equipped with liquid nitrogen-cooled cryo-stages, enabling low-temperature imaging (LT-SEM). To maintain cryogenic temperatures and vacuum conditions, the samples were handled using a proprietary transfer system.

One drop of each sample was smeared on one half of a copper plate. The copper plate was then immersed in liquid nitrogen to fix and freeze the sample. To maintain a clean, visible surface, the samples were then exposed to a partial vacuum in the slushing chamber to solidify the liquid nitrogen. The plate was then transferred (in vacuo) onto a *−*140 °C cryo-stage inside of a Quorum preparatory chamber. The cryo-stage contains a heating element that warms the unit up to *−*90 °C for 10 min, which causes the solid nitrogen and ice to sublimate, thereby exposing any occlusion bodies trapped inside of the frozen liquid. Following in vacuo sublimation, the entire plate was sputter-coated with platinum inside the preparatory chamber. The sample was then transferred into the main body of the LT-SEM onto the central cryo-stage, which was maintained at a constant temperature of *−*140 °C.

Images were taken using both 5 kV and 15 kV accelerating voltages with both low and high probe currents, with either 16 or 64 *s* for each integration, depending on the image resolution (either 1280 p or 5420 p). For each location, images from the upper, middle, and lower detectors were all taken simultaneously and combined into a fourth “mixed” image.

#### 2.2.2. TEM

The transmission electron microscopy of PlidGV-2683 OBs was carried out as previously described [18], except for sections of fixed, embedded OBs, which were stained with 4% uranyl acetate for 10 min after being mounted on copper grids.

### 2.3. DNA Isolation and Sequencing

Aliquots of the PlidNPV-2680 and PlidGV-2683 OB suspensions (500 µL each) were subjected to microcentrifugation, and the pelleted OBs were dissolved in 0.1 M Na_2_CO_3_ as previously described [19]. The dissolved OB solutions were neutralized by the addition of 1/10× volume of 1 M Tris-HCl pH 8.0, and insoluble material was pelleted by microcentrifugation at 4584× *g* for 2 min. The supernatant was transferred to a fresh Eppendorf tube. DNA was extracted and precipitated as previously described [19] and resuspended in deionized, distilled H_2_O. The DNA was quantified with the Quant-iT™ PicoGreen™ dsDNA Assay Kit (Thermo Fisher Scientific, Waltham, MA, USA, #P7589) and a QuantiFluor ™-ST Fluorometer (Promega, Madison, WI, USA). The extraction yielded 472 ng of DNA from the PlidNPV-2680 sample and 2202 ng of DNA from the PlidGV-2683 sample.

Paired-end libraries were prepared from 100 ng of the DNA samples using the KAPA HyperPlus Kit (KK8512), followed by purification and size selection with KAPA Pure Beads (KK8000) following the manufacturer’s instructions. One to two picomoles of each library were sequenced on a MiSeq System (Illumina) using the MiSeq^®^ Reagent Kit v2 micro 300 cycles kit (MS-103-1002). The trimming and assembly of sequencing reads into a consensus sequence and identification of polymorphic positions with insertions, deletions, and variant nucleotides occurring at a frequency of ≥10% were carried out with Lasergene NGen v. 16 (DNAStar, Madison, WI, USA).

### 2.4. Genome Annotation

ORFs ≥ 50 codons in length were annotated if they encoded amino acid sequences with significant sequence similarity by BLASTx against the GenBank nr database with other baculovirus ORFs or genes from other sources. ORFs with no sequence similarity detected by BLASTx were also annotated if (a) they did not occur within the repeat region, (b) they did not overlap a larger ORF by >75 bp, and (c) they were predicted by either FGENESV (http://www.softberry.com/berry.phtml?topic=virus0&group=programs&subgroup=gfindv; accessed on 9 March 2023) or GeneMarkS [20] to be protein-encoding sequences. ORFs with no match in a BLASTx query were used in HMM-HMM comparison queries with HHpred [21] against Pfam-A, UnitProt-SwissProt, and NCBI Conserved Domain databases. The first nucleotide of the polyhedrin (*polh*) or granulin (*gran*) ORFs was set as the first nucleotide in the genome sequence, and downstream annotated ORFs were numbered accordingly.

Baculovirus repeat regions, either homologous regions (*hr*s) or direct repeat regions (*dr*s) [22], were identified in the PlidNPV-2680 and PlidGV-2683 genome sequences with a combination of REPuter [23] and the pattern-finding function of Lasergene GeneQuest 17 (DNASTAR). The presence of potential tRNA genes was evaluated with ARAGORN [24].

Sequence-read archives for these viruses have been submitted under BioProject PRJNA1150071. The annotated PlidNPV-2680 and PlidGV-2683 genome sequences were deposited in GenBank with the accession numbers OQ658191 and PP449363, respectively.

### 2.5. Phylogeny

Baculovirus core gene amino acid alignments were prepared using sequences from PlidNPV-2680 and PlidGV-2683, exemplar viruses of currently classified baculovirus species, and selected other baculoviruses with MUSCLE [25], as implemented in Lasergene MegAlign Pro v. 17.4.2. The core gene alignments were concatenated with BioEdit 7.2.6 [26], and phylogeny was inferred by maximum likelihood (ML) using RAxML [27] from the concatenated core gene alignments with the Le and Gascuel (LG) substitution matrix [28] and variable rates among sites, empirical amino acid frequencies, and 100 rapid bootstrap replicates.

Amino acid sequences specified by PlidNPV-2680 and PlidGV-2683 ORFs encoding Inhibitor-of-Apoptosis Protein (IAP) homologs were aligned with other baculovirus and lepidopteran IAP homologs by MAFFT [29], as implemented in Lasergene MegAlign Pro v. 17.4.2, and phylogenies were inferred by ML using MEGA11 [30] and the LG substitution matrix with a gamma distribution parameter of 1.63.

### 2.6. Gene Synteny and Pairwise Distance Estimation

Gene synteny between PlidNPV-2680 and selected members of the genus *Alphabaculovirus* was assessed by gene parity plot analysis as previously described [31]. Pairwise nucleotide distances between PlidNPV, PlidGV, and exemplar viruses of species in the genera *Alphabaculovirus* or *Betabaculovirus* were estimated from alignments of partial *lef-8*, *lef-9*, and *polh* sequences using the Kimura-2-parameter substitution matrix with gamma parameters estimated using MEGA11, as previously described [32].

## 3. Results

### 3.1. Ultrastructure Features of P. idaeusalis Baculovirus Occlusion Bodies

**Figure 1:**
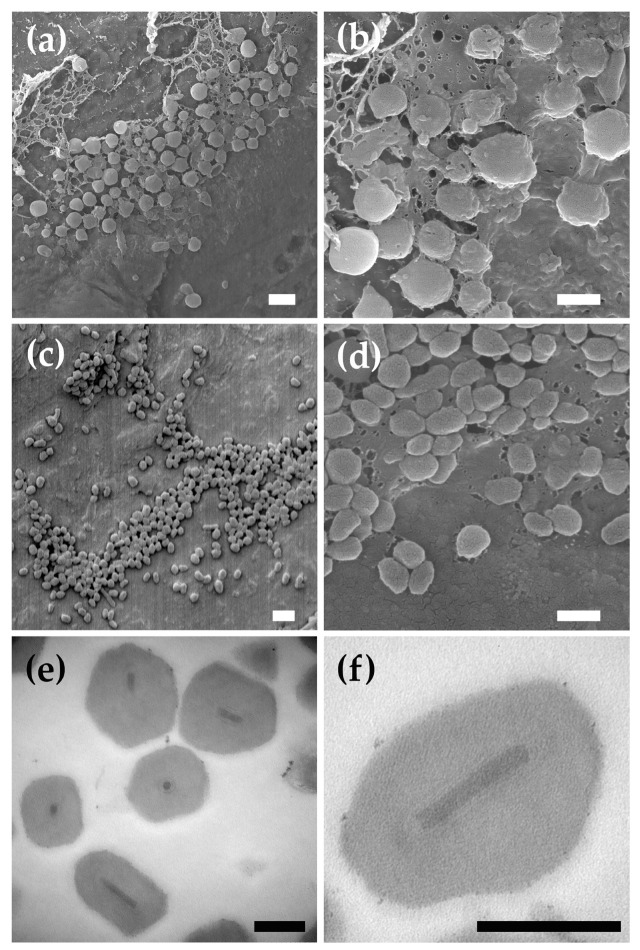
OBs of an alphabaculovirus and betabaculovirus isolate from the tufted apple bud moth, *P. idaeusalis*. (**a**,**b**) Scanning electron micrographs of OBs from alphabaculovirus PlidNPV-2680. (**c**,**d**) Scanning electron micrographs of OBs from betabaculovirus PlidGV-2683. (**e**,**f**) Micrographs of ultrathin sections through PlidGV-2683 OBs. Scale bars: (**a**) 2 μm; (**b**,**c**) 1 μm; (**d**) 0.5 μm; and (**e**,**f**) 0.2 μm.

Previously published transmission electron micrographs of PlidNPV-2680 OBs revealed a typical alphabaculovirus virion structure consisting of multiple rod-shaped nucleocapsids per unit envelope [14]. PlidNPV-2680 OBs exhibited an irregular polyhedral shape typical of alphabaculovirus OBs in scanning electron micrographs (Figure 1a,b), with an average diameter of 0.94 +/−0.027 μm (n = 57).

PlidGV-2683 OBs were distinguished by a roughly ovocylindrical shape typical of betabaculovirus OBs (Figure 1c,d). Betabaculovirus OBs have been reported to be approximately 0.12 μm in width [5,33], but PlidGV-2683 OBs were more than twice as wide, with dimensions measuring 0.27+/*−*0.007 μm in width and 0.43+/*−*0.006 μm in length (n = 41). Sections through PlidGV-2683 OBs revealed a single enveloped virion per OB (Figure 1e,f).

### 3.2. Properties of P. idaeusalis Alphabaculovirus and Betabaculovirus Genomes

Sequencing reads for PlidNPV-2680 and PlidGV-2683 were assembled into circular contigs of 121,881 and 106,633 bp, respectively (Table 1), with 261- and 71-fold coverage, respectively. Nucleotide distributions (%G + C) differed between the two sequences by 7.85%. Although the PlidNPV genome was >15 kbp larger than the PlidGV genome, similar numbers of ORFs were annotated for the two viruses (128 for the NPV and 125 for the GV; Tables S1 and S2).

**Figure 2:**
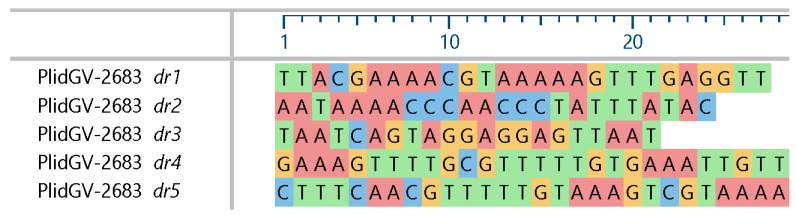
Consensus sequences of unit repeats in the direct repeat (*dr*) regions of PlidGV-2683. The consensus sequences for each repeat region (labeled *dr*#) are shown, unaligned, with nucleotide-specific color shading.

Screening the genome sequences for repeat regions revealed that the PlidNPV genome contained 10 *hrs*, each consisting of two-to-seven 44 bp imperfect palindromic repeats that were conserved among the *hr*s with the consensus sequence 5′-CCTAAATGGAATTCATTACCGAATGTAAATGGAGCCAGTTTGGA-3′. In contrast, an examination of the PlidGV genome sequence did not reveal any hrs. Instead, five intergenic regions were found to contain direct repeats of short sequences that differed from one region to the next (Figure 2). These regions were designated as *dr*s and were numbered consecutively based on their order in the genome annotation. The *dr*s possessed six (*dr1*, *dr2*, *dr5*) to eleven (*dr4*) or twelve (*dr3*) copies of their respective repeats.

Putative transfer RNA (tRNA) genes have been identified in some baculovirus genomes [34], but the examination of the PlidNPV and PlidGV genome sequences with ARAGORN did not detect the presence of tRNA genes in either sequence.

The PlidNPV-2680 sequence was relatively homogenous, with only three single-nucleotide polymorphisms (SNPs) that occurred at frequencies ≥10%, ranging from 10.2% to 18.5% of the reads covering the positions (Table S3). The SNPs occurred in ORFs, and the primary variant nucleotides at each site were non-synonymous substitutions. The variant analysis also pointed to the presence of a 280 bp deletion, present at a frequency of approximately 12%, which removed most of hr8. The PlidGV-2683 sequence was more heterogeneous, with 441 polymorphic sites containing variants present at frequencies ≥10%, including 20 indels and 421 SNPs (Table S3). All but one of the indels was 1 bp in size. The indels ranged in frequency from 12.5 to 50.0%, and two occurred within ORFs. The SNP frequencies ranged from 10% to 47.36%, with 370 occurring within ORFs, 11 occurring with *dr*s, and 39 occurring within intergenic regions.

### 3.3. ORF Content

#### 3.3.1. Conserved Baculovirus Genes

**Figure 3:**
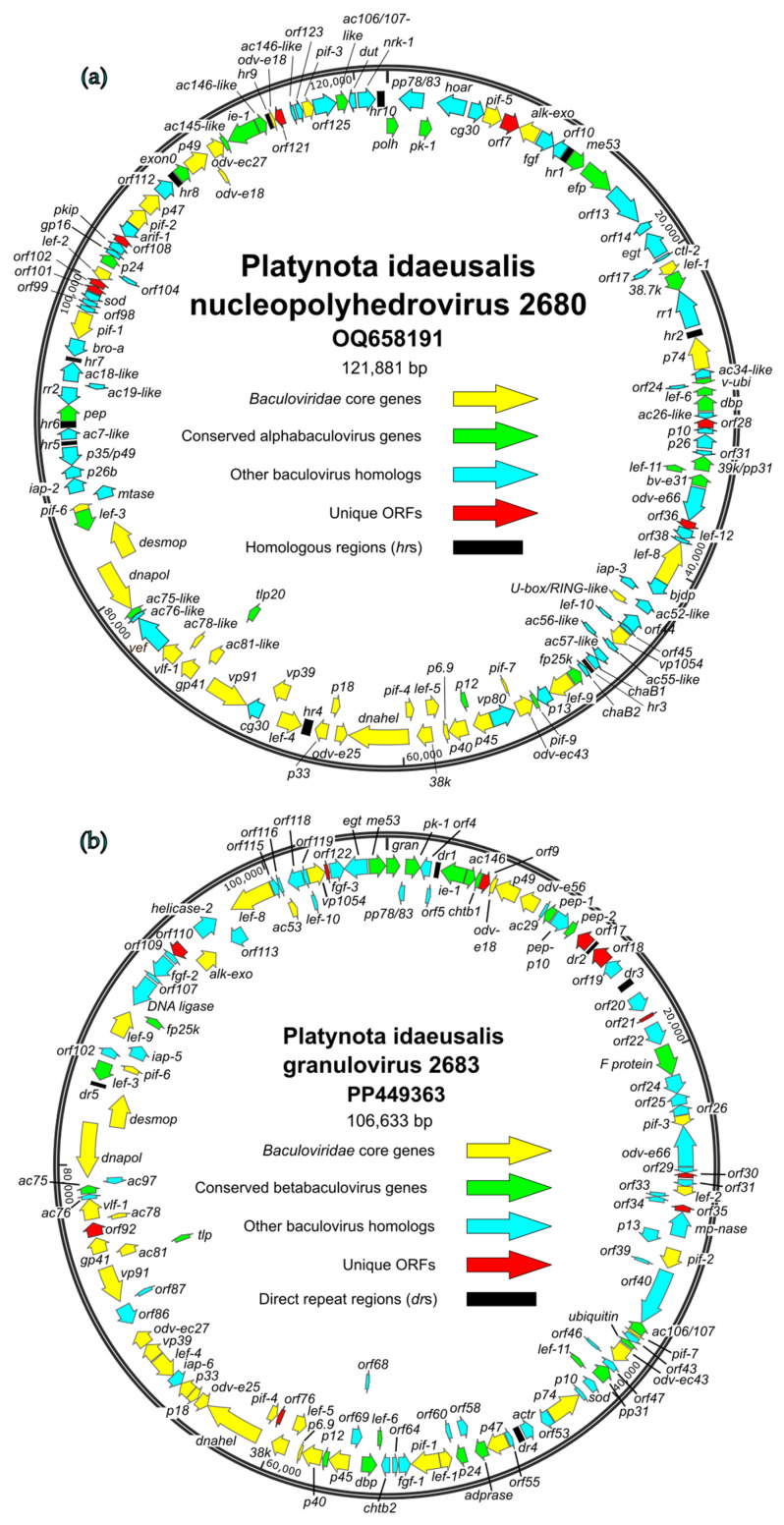
Maps of the open reading frames (ORFs) and other features of (**a**) PlidNPV-2680 and (**b**) PlidGV-2683. ORFs are represented by arrows, with the position and direction of the arrow indicating the ORFs’ position and orientation. Each ORF is color-coded to indicate whether it corresponds to a baculovirus core gene conserved in all four genera of *Baculoviridae* (yellow; [10,11]), an ORF conserved only among alphabaculoviruses or betabaculoviruses (green, [10]), an ORF with homologs in a more limited subset of other baculoviruses (blue), or an ORF with no baculovirus homologs (red). Homologous regions (*hr*s) or direct repeat regions (*dr*s) are represented by black rectangles. ORFs are designated by either the names by which they are referred to in the literature or a number corresponding to their annotation in the genome.

Both the PlidNPV-2680 and PlidGV-2683 genomes possessed a full complement of the 38 core genes that define the family Baculoviridae [10,11] (Figure 3 (yellow arrows), Tables S1 and S2). Garavaglia and co-workers listed an additional 26 genes that were detected in all of the 58 alpha- and betabaculovirus genome sequences available for analysis at the time [10], and genes in this category are indicated in Figure 3 with green arrows. Of this second set of genes, gp37 (ac64) was missing from both genomes, and 38.7k (ac13) and exon0 (ac141) were also missing from the sequence of PlidGV-2683. Also of note, both PlidNPV-2680 and PlidGV-2683 lacked the chiA (ac126) and v-cath (ac127) genes, which encoded a chitinase and a cathepsin L cysteine protease, respectively.

The baculovirus genes dbp (ac25; [35]), fgf (ac32; [36]), and p26 (ac136; [37]) occurred in multiple copies in many baculovirus genomes. Two copies of p26 were identified in PlidMNPV-2680, while three copies of fgf were found in PlidGV-2683. Baculovirus genes also often include members of the baculovirus-repeated ORF (bro) multigene family [38]. PlidNPV-2680 was found to have a single bro ORF, while none were detected in PlidGV-2683. The Inhibitor-of-Apoptosis Protein (IAP) family is another prominent baculovirus multigene family, the members of which encode proteins that, in some cases, can regulate the host’s apoptotic response to baculovirus infection [39]. These genes have been found to occur in multiple distinct lineages. PlidNPV-2680 contained one *iap* each of the *iap-1* and *iap-2* lineages, while PlidGV-2683 contained one *iap* each of the *iap-5* and *iap-6* lineages.

Finally, PlidNPV-2680 was found to contain a single copy of an enhancin gene. This gene encodes a zinc metalloprotease homolog that facilitates oral infectivity [40] and is often present in multiple copies in those baculovirus genomes where it occurs [41,42]. In studies with Lymantria dispar multiple nucleopolyhedrovirus, both copies of its enhancin gene were found to contribute to virulence against its larval host, and both copies were required to produce the level of virulence typically observed with this virus [43].

#### 3.3.2. Unique Genes

Of the ORFs annotated for the two *P. idaeusalis* baculovirus genomes, six of the ORFs in PlidNPV-2680 and nine of the ORFs in PlidGV-2683 did not possess homologs in other baculovirus genomes identifiable by BLAST (Table 2). The ORFs listed in Table 2 were unique to either PlidNPV-2680 or PlidGV-2683, with the exception of PlidNPV ORF121 and PlidGV ORF9, which are homologs of each other (see Section 3.3.3). Analysis with HHpred identified U-box/RING-like and RING finger domains in four of these ORFs. PlidNPV ORF121 and PlidGV ORF9 were found to contain a U-box/RING-like domain observed in the baculovirus core gene *ac53* [10]. PlidGV ORFs 17 and 18 were observed to have N-terminal RING domains similar to those found in the baculovirus genes *ac88* (*cg30*; [44]), *ac151* (*ie2*; [45]), and *ac153* (*pe38*; [46]). ORF17 and ORF18 occurred on either side of the *dr2* repeat element in the PlidGV sequence and appeared to be duplicates of each other, sharing 49% overall sequence identity and identical 32-amino acid leader sequences.

A BLASTx query with the 246-amino acid sequence encoded by PlidGV-2683 ORF92 yielded matches with significant sequence similarity to lepidopteran IAPs but no matches with baculovirus IAPs. Closer scrutiny of the sequence revealed that the ORF92 sequence contains two Baculoviral Inhibitor of apoptosis Repeat (BIR) domain sequences, but the first BIR sequence is preceded by a leader sequence of only 12 amino acids that lacks the cleavage and destabilization motifs associated with cellular IAPs [47,48]. The phylogenetic inference of the relationships of ORF92 with other lepidopteran and baculovirus IAPs placed ORF92 in a clade with baculovirus IAP-3 homologs (Figure S1).

#### 3.3.3. ORFs in PlidNPV-2680 That Appear to Derive from Betabaculoviruses

**Figure 4:**
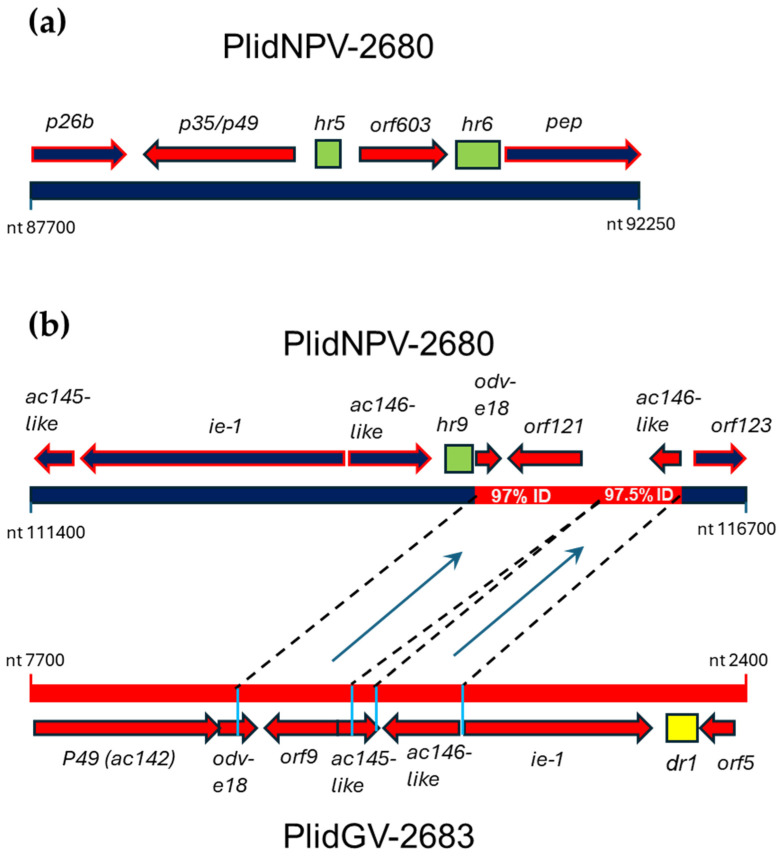
Betabaculovirus homologs present in the alphabaculovirus PlidNPV-2680 genome. (**a**) Representation of the region in PlidNPV from nucleotide positions 87,700 to 92,260 showing betabaculovirus homologs of *p35/p49* and *orf603* (*ac7*). (**b**) Representation of the acquisition of PlidGV sequences containing three ORFs by PlidNPV. Genome segments and ORFs of PlidNPV-2680 are in dark blue, while segments and ORFs of betabaculovirus origin are in red. Dashed lines and light blue vertical lines denote the regions of PlidGV that are present in the PlidNPV-2680 genome, and the nucleotide sequence identities with the acquired PlidGV sequences are indicated (% ID).

The PlidNPV-2680 genome sequence contains two regions with ORFs that exhibit top matches with betabaculovirus ORFs using BLASTx (Figure 4) rather than with alphabaculovirus ORFs. In the first region, PlidNPV ORF90 was found to encode a 369-amino acid sequence with BLAST matches to betabaculovirus homologs of the P35/P49 inhibitor of apoptosis (*ac135*), exhibiting 34.2% sequence identity to the Choristoneura fumiferana granulovirus P35/P49 homolog (GenBank accession no. YP_654436; [49]; Figure 4a andexemplar alphabaculoviruses measured Table S1). ORF91 encoded a 214 amino-acid sequence with 45.5% sequence identity to the Clostera anastomosis granulovirus B Clas51 ORF, which is a betabaculovirus homolog of the AcMNPV ORF *ac7* (*orf603*; Figure 4a, Table S1). These two ORFs were both adjacent to *hr*s and flanked by alphabaculovirus ORFs (Figure 4a).

The second region was found to contain three ORFs with top BLAST matches for betabaculovirus homologs downstream of *hr9* (Figure 4b). A comparison with the nucleotide sequence of PlidGV-2683 revealed that a 1516 bp region of PlidNPV-2680 containing these betabaculovirus homologs exhibited 97.5% sequence identity to PlidGV-2683 nt 4510–5158 and 97% sequence identity to PlidGV-2683 nt 5330–6189. No other PlidGV-2683 sequences were identified in or assembled from the sequencing reads of the PlidNPV-2680 sample.

The PlidGV ORFs present in PlidNPV-2680 included the C-terminal portion of PlidGV *odv-e18*, all of PlidGV ORF9, and a C-terminally truncated version of PlidGV *ac146*. Only the first 31 codons of PlidGV *ac145* (located between ORF9 and *ac146*) were transferred to PlidNPV, so this sequence was not annotated in the PlidNPV-2680 genome. The copy of *odv-e18* transferred from PlidGV was missing the N-terminal amino acids encoded by the PlidGV ORF, including a predicted transmembrane domain that may be required for the incorporation of ODV-E18 into the occlusion-derived virus membrane [50]. The start codon for this *odv-e18* ORF was 32 bp downstream of *hr9*. In the *ac146* ORF transferred from PlidGV, a frameshift in the 72nd codon led to the occurrence of a stop codon that resulted in a 113-amino acid deletion in the predicted gene product of the PlidNPV-2680 homolog. An intact PlidGV ORF9 sequence was present in PlidNPV-2680, designated as ORF121 in the PlidNPV annotation. The PlidGV and PlidNPV amino acid sequences of this ORF shared 90.5% sequence identity. Homologs for this ORF were not identified in other baculoviruses, but a baculovirus U-box/RING-like domain was identified in the C-terminal sequence by HHpred with >97.5% probability (Table 2). No contigs were produced from attempts to assemble PlidGV-2683 sequencing reads on a PlidNPV-2680 template (and vice versa), indicating that the PlidNPV-2680 sample did not contain PlidGV-2683 (and vice versa).

### 3.4. Relationships with Other Viruses

**Figure 5:**
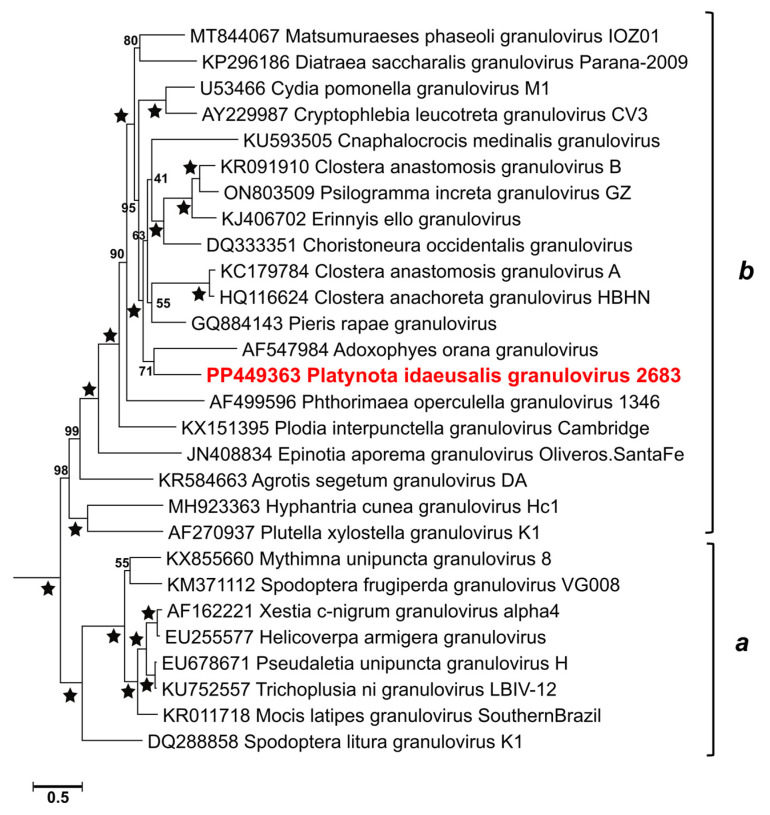
Phylogeny showing the relationship of PlidGV-2683 to other viruses of the genus *Betabaculovirus*. The betabaculovirus subtree of a phylogram inferred from concatenated MUSCLE alignments of baculovirus core gene amino acid sequences by maximum likelihood is shown. Taxon names and genome sequence GenBank accession numbers are indicated for each branch, with PlidGV-2683 in red bold type. Betabaculovirus clades (**a**,**b**) [51] are indicated with brackets. The levels of bootstrap support for each node are shown, with 100% bootstrap support denoted by stars.

BLASTx queries with ORFs of PlidNPV and PlidGV clearly indicated the likely genera for these viruses but did not point to specific alpha- or betabaculoviruses that were closely related to them. Core gene phylogeny placed PlidGV-2683 in clade b of the genus Betabaculovirus [51] and in a node with the Adoxophyes orana granulovirus with 71% bootstrap support (Figure 5).

**Figure 6:**
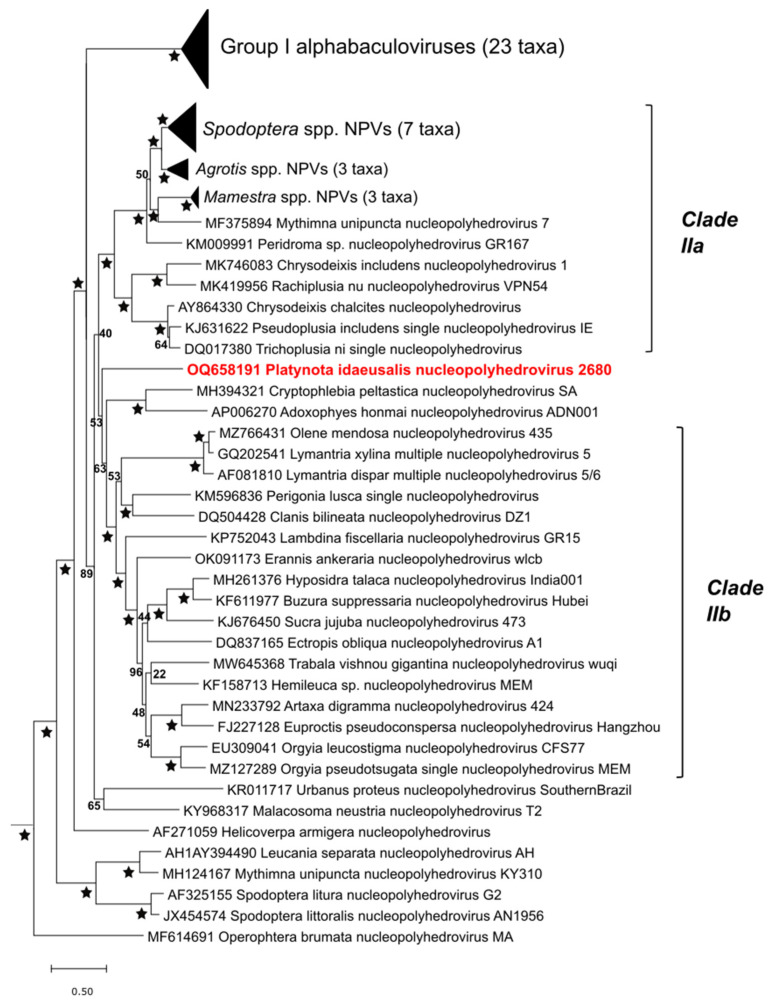
Phylogeny showing the relationship of PlidNPV-2680 to other viruses of the genus *Alphabaculovirus*. The alphabaculovirus subtree of a core gene phylogram, which was inferred as described for the betabaculovirus subtree (Figure 5), is shown. Taxon names and genome sequence GenBank accession numbers are indicated for each branch, with PlidNPV-2680 in red bold type. Alphabaculovirus group II clades IIa and IIb are indicated with brackets. The branches for groups of related viruses are collapsed, with the number of taxa indicated for each collapsed set. The levels of bootstrap support for each node are shown, with 100% bootstrap support denoted by stars.

The same analysis placed PlidNPV among group II alphabaculoviruses (Figure 6). Viruses in this group were missing the gp64 envelope fusion gene associated with group I alphabaculoviruses, and a prior comprehensive phylogenetic analysis divided these viruses into three clades: clade IIa, clade IIb, and clade IIc [52]. The viruses of clade IIc were not recovered as a monophyletic group in the analysis shown in Figure 6. PlidNPV-2680 was placed on a branch in a poorly supported node that included clade IIb viruses as well as Cryptophlebia peltastica nucleopolyhedrovirus SA and Adoxophyes honmai nucleopolyhedrovirus ADN001.

**Figure 7:**
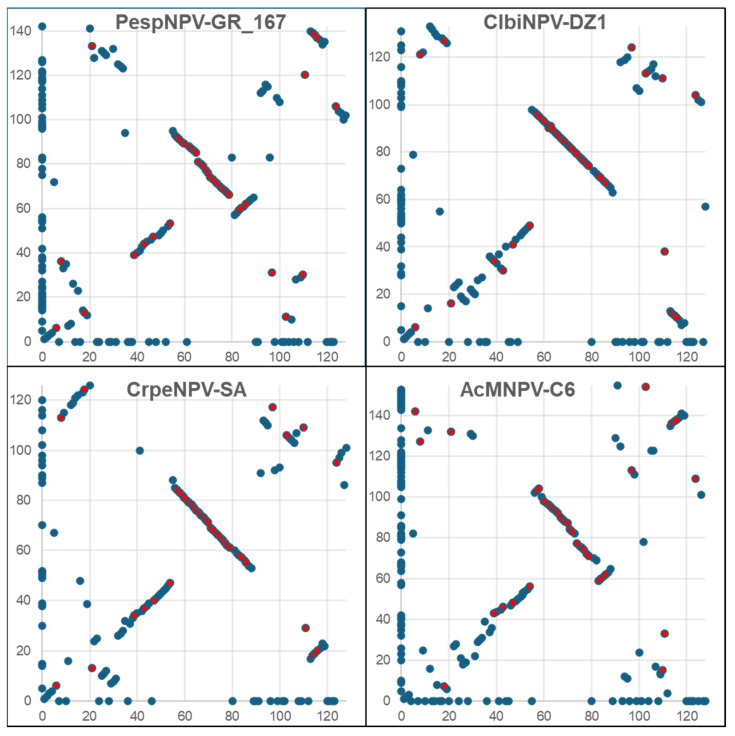
Gene-parity plots comparing the ORF content and order of PlidNPV-2680 (x-axis) with four other alphabaculoviruses (y-axis), including representatives of group I (Autographa californica multiple nucleopolyhedrovirus, AcMNPV-C6), clade IIa (Peridroma sp. nucleopolyhedrovirus GR167 and PespNPV-GR167), clade IIb (Clanis bilineata nucleopolyhedrovirus DZ1 and ClbiNPV-DZ1), and clade IIc (Cryptophlebia peltastica nucleopolyhedrovirus SA and CrpeNPV-SA). Points correspond to individual ORFs. ORFs occurred only in one of the two genomes being compared and are plotted directly on the axis corresponding to the virus containing the ORF. Red points denote baculovirus core genes.

Gene synteny between PlidNPV-2680 and representatives of group I, clade IIa, clade IIb, and clade IIc was assessed by gene parity plot analysis (Figure 7, [31]). All four comparisons featured a block of genes, including several baculovirus core genes with conserved synteny. This conserved block has been previously reported [53,54] and is a common feature of baculovirus gene parity plots. Although other blocks of ORFs with conserved synteny were clearly evident in individual plots, no obvious pattern connecting PlidNPV-2680 to one of the other groups of alphabaculoviruses was evident from this analysis.

Kimura-2-parameter pairwise nucleotide distances in the conserved regions of *lef-8*, *lef-9*, and *polh* between PlidNPV-2680 and exemplar alphabaculoviruses measured ≥0.73 (*lef-8*), ≥0.38 (*lef-9*), and ≥0.41 (*polh*) substitutions/site, which is well above the 0.05 substitutions/site criterion for the demarcation of baculovirus species [32]. Pairwise distances between PlidGV-2683 and exemplar betabaculoviruses at the same loci measured ≥0.79 (*lef-8*), ≥0.68 (*lef-9*), and ≥0.33 (*polh*) substitutions/sites. These results, together with core gene phylogeny and gene synteny, collectively indicate that both PlidNPV-2680 and PlidGV-2683 represent new species of baculoviruses.

## 4. Discussion

Homologous recombination between closely related baculoviruses can occur frequently during co-infection and replication [55,56]. Gene transfer between more divergent baculoviruses infecting the same host appears to occur far less frequently [57], but instances of horizontal gene transfer have been identified between more distantly related groups of insect dsDNA viruses [58]. An examination of the current ICTV species lists for the genera *Alphabaculovirus* and *Betabaculovirus* indicates that there are at least eight host lepidopterans from which both an alphabaculovirus and a betabaculovirus have been isolated. Differences in the kinetics, tissue trophism, and cytopathology of infection and replication by alphabaculoviruses and betabaculoviruses [33,59,60] may pose barriers to exchanges of genetic material during the co-infection of larvae. However, homologs of individual betabaculovirus ORFs have been observed in some alphabaculovirus genomes [18,61,62], supporting the idea that such exchanges occur, as well as acquisitions of alphabaculovirus genes by betabaculoviruses [63]. A previous analysis of the genome of the Mamestra configurata nucleopolyhedrovirus B isolate 96B (MacoNPV-B 96B) revealed a more extensive recombination event, similar to that documented for PlidGV and PlidNPV, in which a 5.4 kbp region of the genome shared ≥95% nucleotide sequence identity with two adjacent segments from the Xestia c-nigrum granulovirus (XecnGV) genome sequence [64]. MacoNPV-B 96B contained complete homologs of XecnGV ORFs 61 and 65 and truncated homologs of the XecnGV ORFs 60, 62, and 64 as a consequence. This 5.4 kbp region was found to be missing from other isolates of MacoNPV-B [65], suggesting that the acquisition of this sequence from XecnGV occurred recently and MacoNPV-B viruses with this genotype had not spread to European populations of MacoNPV-B or the closely related Mamestra brassicae nucleopolyhedrovirus (MabrNPV). It is possible that the acquisition of the PlidGV sequence by PlidNPV is similar in terms of occurring relatively recently, and isolates with the PlidNPV-2680 genotype may not be widespread among PlidNPV populations, although this remains to be confirmed.

The homologs of the betabaculovirus ORFs present in PlidNPV-2680 occur adjacent to *hr*s (Figure 4). A high degree of variability in the ORF content around *hr*s in baculovirus genomes has been observed [66], indicating that they are sites for genomic recombination. The presence of AcMNPV *hr5* doubled the incidence of recombination in a recombination assay [67]. Several studies have pointed to *hr*s acting as origins of baculovirus DNA replication [68,69,70,71], and the replication of two different baculoviruses in the same host cell has been associated with a high frequency of recombination between them during infection [56]. The region in PlidNPV-2680 containing a homolog of PlidGV-2683 *ac146* occurred immediately downstream of PlidNPV *hr9*, which, in turn, was flanked by the native PlidNPV *ac146* ORF (Figure 4b). It is conceivable that during a co-infection of host cells by PlidNPV and PlidGV, the unwinding of the PlidNPV DNA duplex in the region starting at *hr9* may have been followed by strand exchange with replicating PlidGV somewhere within the *ac146* sequences of the viruses, followed by the resolution of the resulting recombination intermediate that placed the PlidGV sequence downstream of *hr9* in PlidNPV. Although the overall nucleotide sequence identity between PlidNPV and PlidGV in this region is low (approximately 50% identity), it may be possible for a rare recombination event to occur in baculovirus-infected cells even with a short stretch of homologous base-pairing or the presence of several mismatches [72,73].

The copies of PlidGV *odv-e18* and *ac146* present in PlidNPV-2680 were found to be both truncated and seem unlikely to encode active ODV-E18 and AC146 proteins. An intact copy of PlidGV ORF9 was present in the PlidNPV-2680 genome with 90.5% sequence identity and a conserved U-box/RING-like domain, suggesting that the activity of the protein may be preserved in the PlidNPV-2680 copy. Members of the baculovirus P35/P49 gene group encode inhibitors of apoptosis [74], and the intact betabaculovirus copy of *p35/p49* in PlidNPV-2680 may provide a new countermeasure against a larval host apoptotic response to infection. The function of ORF603 (AC7) is unknown, but a knockout mutation in its ORF in Autographa californica multiple nucleopolyhedrovirus [75] resulted in reduced survival time in *Spodoptera frugiperda* larvae in bioassays, suggesting that its appearance in PlidNPV-2683 may affect viral virulence.

The placement of PlidGV-2683 in a clade with AdorGV [76] is consistent with the co-evolution of baculoviruses with their hosts, as the hosts of these two betabaculoviruses are both moths of the family Tortricidae. However, PlidGV-2683 is distinguished by the absence of *38.7k* (*ac13*). While several betabaculoviruses have been found to be missing gp37 and exon0 [77,78,79,80,81], the *38.7k* gene appears to be conserved among both alpha- and betabaculoviruses. Studies with *38.7k* knockout mutants of Bombyx mori nucleopolyhedrovirus and Autographa californica multiple nucleopolyhedrovirus have revealed that the 38.7K protein is associated with the host nuclear envelope and is involved in the production of both occluded and budded virions [82,83,84].

The alphabaculovirus phylogeny suggests that PlidNPV-2680 represents a previously undetected lineage in the genus Alphabaculovirus. This observation is underscored by the absence of *chiA* and *v-cath*. The products of these genes act in concert to liquefy the internal anatomy of infected larvae and weaken the cuticle to the point that it ruptures post-mortem, thus promoting the spread of infectious OBs [12,85,86]. These genes are also missing from the PlidGV-2683 genome, but while several betabaculoviruses have been discovered not to have these two genes, only four alphabaculoviruses have been reported to be missing both genes [87,88,89,90], with a fifth missing only the chitinase gene [91].

At the time of writing this article, there are currently 97 species listed for the family *Baculoviridae* on the ICTV website. This number rivals or exceeds the current numbers of species listed for other significant families of eukaryotic DNA viruses, such as *Adenoviridae* (109 species), *Orthoherpesviridae* (118 species), and *Poxviridae* (83 species). The baculovirus genomes reported in this paper add to our accumulated knowledge of the baculovirus and its genetic and genomic diversity while providing an example of recombination between viruses that share a host, hinting that a greater degree of diversity remains to be discovered and described.

## Figures and Tables

**Table 1:** Properties of *P. idaeusalis* baculovirus genomes.

Isolate	Size (bp)	%GC	Annotated ORFs	Repeated Regions	GenBank Accession No.
PlidNPV-2680	121,881	39.22%	128	10 *hr*s	OQ658191
PlidGV-2683	106,633	31.37%	125	5 *dr*s	PP449363

**Table 2:** ORFs in *P. idaeusalis* baculovirus genomes with no baculovirus homologs.

Isolate	ORF	Nt Position	Size (aa)	BLAST/HHpred Query Results
PlidNPV-2680	7	7197 → 8261	354	-
	28	30,507 ← 31,052	181	-
	36	36,833 → 37,321	162	-
	101	99,237 → 99,662	141	-
	108	102,642 → 103,100	152	-
	121	114,940 ← 115,479	179	Baculovirus U-Box/RING-like domain, residues 122–167; HHpred probability 97.3%.
PlidGV-2683	9	5428 → 5964	178	Baculovirus U-Box/RING-like domain residues 121–166; HHpred probability 97.51%.
	17	11,791 ← 12,639	282	RING finger domains in baculovirus CG30, IE2, PE38, residues 3–58, top HHpred probability 99.2%
	18	13,005 ← 14,015	336	As for ORF17.
	21	17,812 → 17,979	55	-
	30	27,087 ← 27,341	84	-
	35	28,907 ← 29,260	117	Zinc finger domains in reovirus outer capsid protein and AcMNPV CG30, HHpred probabilities 94.5–94.9%.
	76	59,983 → 60,270	95	-
	92	75,968 → 76,708	246	baculoviral IAP repeat-containing protein 3 isoform X2 [Nymphalis io], 49.9% identity, e = 3.9 × 10^−100^.
	110	93,221 ← 93,871	216	Putative F-box proteins, residues 1–35, top HHpred probability 92.9% with putative F-box/LRR-repeat protein R542 from Acanthamoeba polyphaga mimivirus

## Data Availability

The original data presented in the study are openly available within the NCBI BioProject repository at https://www.ncbi.nlm.nih.gov/bioproject/?term=PRJNA1150071 (accessed on 16 December 2024). Annotated PlidNPV-2680 and PlidGV-2683 genome assemblies are available at GenBank under accession numbers OQ658191 and PP449363, respectively.

## Supplementary Materials

The following supporting information can be downloaded at https://www.mdpi.com/article/10.3390/v17020202/s1. Figure S1: Phylogeny of IAP amino acid sequences from baculoviruses and lepidopterans; Table S1: PlidNPV-2680 open reading frames (ORFs) and homologous repeat regions (*hr*s); Table S2: PlidGV-2683 open reading frames (ORFs) and direct repeat regions (*dr*s); Table S3: SNPs and indels.
