# Supplementary material for: The Genome Sequences of Baculoviruses from the Tufted Apple Bud Moth, Platynota idaeusalis, Reveal Recombination Between an Alphabaculovirus and a Betabaculovirus from the Same Host
Source: Viruses. 2025 Jan 30;17(2):202. doi: 10.3390/v17020202 (PMC11861948; doi:10.3390/v17020202)
Supplement: Supplementary file 1 [file viruses-17-00202-s001.zip › Figure S1.pdf]

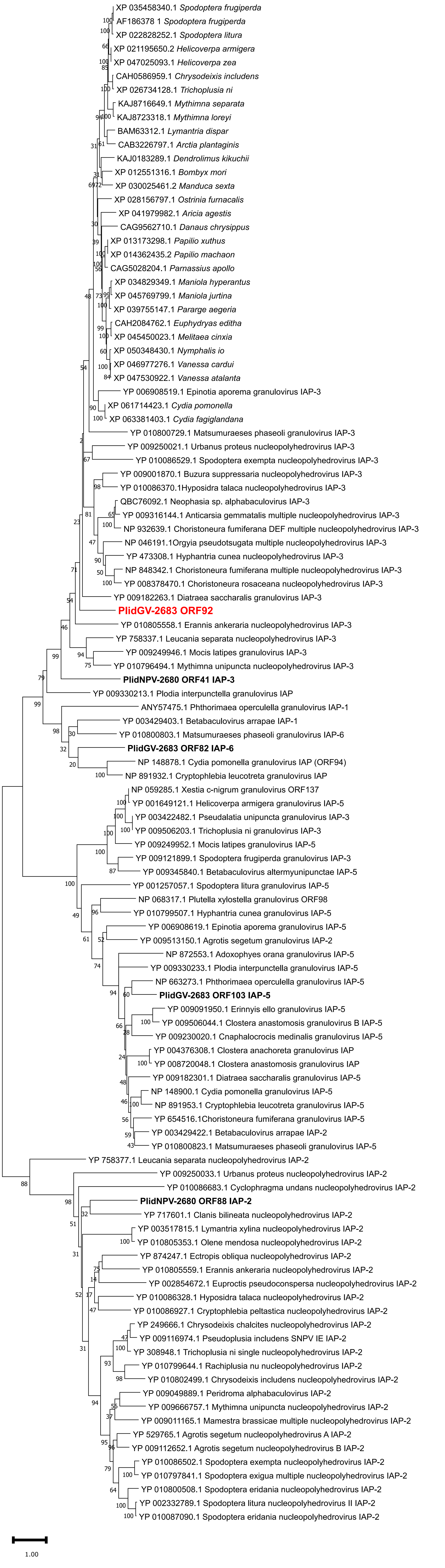

Figure S1. Phylogeny of IAP amino acid sequences from baculoviruses and lepidopterans. IAP sequences were aligned by MAAFT ass implemented in LaserGene MegAlign Pro 17, and phylogeny was inferred by maximum likelihood in MEGA11 using the Le_Gascuel_2008 model with a gamma distribution parameter of 1.63. PlidGV-2683 ORF92, which matched only with lepidopteran IAP sequences in a BLASTx query, is shown in red bold type. The other IAP sequences identified in PlidNPV-2680 and PlidGV-2683 are shown in black bold type.
