# Supplementary material for: The Genome Sequences of Baculoviruses from the Tufted Apple Bud Moth, Platynota idaeusalis, Reveal Recombination Between an Alphabaculovirus and a Betabaculovirus from the Same Host
Source: Viruses. 2025 Jan 30;17(2):202. doi: 10.3390/v17020202 (PMC11861948; doi:10.3390/v17020202)
Supplement: Supplementary file 1 [file viruses-17-00202-s001.zip › Table S1.pdf]

Table S1. PlidNPV-2680 open reading frames (ORFs) and homologous repeat regions (*hr*s)

ORF	Name	Position	aa	Top BLASTx match	% identity (range of alignment)	Notes/AcMNPV homologs
**1**	*polh*	1→741	246	Polyhedrin [Perigonia lusca single nucleopolyhedrovirus]	93.5 (230/246)	*ac8*
**2**	*pp78/83*	738←2210	490	pp78/83 [Orgyia pseudotsugata single capsid nuclopolyhedrovirus]	27.2 (146/536)	*ac9*
**3**	*pk-1*	2203→2982	259	PK1 [Agrotis segetum nucleopolyhedrovirus A]	60.5% (156/258)	*ac10*
**4**	*hoar*	3069←4799	576	hoar [Trichoplusia ni single nucleopolyhedrovirus]	32 (79/247)	
**5**	*cg30*	5090→5983	297	CG30 [Perigonia lusca single nucleopolyhedrovirus]	40.3 (25/62)	*ac88*
**6**	*pif-5/odv-e56*	6026→7114	362	Per os infectivity factor 5 [Trabala vishnou gigantina nucleopolyhedrovirus]	69.7 (221/317)	*ac148*
**7**		7197→8261	354			
**8**	*alk-exo*	8305←9516	403	Alkaline exonuclease [Perigonia lusca single nucleopolyhedrovirus]	47.1 (193/408)	*ac133*
**9**	*fgf*	9630→10658	342	fibroblast growth factor [Malacosoma neustria nucleopolyhedrovirus]	31.3 (80/256)	*ac32*
**10**		10672←11415	247	hypothetical protein [Spodoptera cosmioides nucleopolyhedrovirus]	37.4 (71/190)	
	hr1	11487 - 11810				5 repeats
**11**	*me53*	11871→12869	332	Major early transcription protein 53 [Trabala vishnou gigantina nucleopolyhedrovirus]	34.3% (110/321)	*ac139*
**12**	*efp*	13013→15064	683	fusion protein [Malacosoma neustria nucleopolyhedrovirus]	39.6 (226/570)	*ac23*
**13**	*peptidase MA*	15155→17635	826	Ld129-like protein [Clanis bilineata nucleopolyhedrovirus]	30.8% (227/738)	
**14**		17800←18342	180	hypothetical protein [Perigonia lusca single nucleopolyhedrovirus]	31.0% (58/187)	
**15**	*egt*	18496←20052	518	ecdysteroid UDP-glucosyltransferase [Trichoplusia ni single nucleopolyhedrovirus]	66.5 (333/501)	*ac15*
**16**	*ctl-2*	20185→20343	52	conotoxin-like protein [Apocheima cinerarium nucleopolyhedrovirus]	67.3 (35/52)	
**17**		20366→20722	118	hypothetical protein EONV_gp123 [Ectropis obliqua nucleopolyhedrovirus]	41.2 (46/112)	
**18**	*lef-1*	20719→21402	227	lef-1 [Cryptophlebia peltastica nucleopolyhedrovirus]	50.0% (114/230)	*ac14*

ORF	Name	Position	aa	Top BLASTx match	% identity (range of alignment)	Notes/AcMNPV homologs
**19**	*38.7k*	21408→22472	354	38.7k [Mamestra brassicae multiple nucleopolyhedrovirus]	45.2 (151/334)	*ac13*
**20**	*rr1*	22519←24834	771	ribonucleotide reductase, large subunit [Euproctis pseudoconspersa nucleopolyhedrovirus]	65.8% (510/775)	
	hr2	25130 - 25453				5 repeats
**21**	*p74*	25519←27468	649	p74 [Hemileuca sp. nucleopolyhedrovirus]	68.6 (460/671)	*ac138*
**22**	*ac34-like*	27484←28014	176	ORF-136 [Agrotis segetum nucleopolyhedrovirus A]	62.9% (105/167)	*ac34*
**23**	*v-ubi*	28086→28382	98	Putative Ubiquitin-60S ribosomal protein L40 [Lichtheimia ramosa]	87.9 (58/66)	*ac35*
**24**		28321→28524	67	ORF-31 [Buzura suppressaria nucleopolyhedrovirus]	44.8 (30/67)	
**25**	*lef-6*	28577←29089	170	lef-6 [Helicoverpa armigera nucleopolyhedrovirus]	57.7% (41/71)	*ac28*
**26**	*dbp*	29099←30034	311	DNA binding protein [Helicoverpa armigera nucleopolyhedrovirus]	45.0 (135/300)	*ac25*
**27**	*ac26-like*	30137→30493	118	ORF-138 [Agrotis segetum nucleopolyhedrovirus A]	44.7% (46/103)	*ac26*
**28**		30507←31052	181			
**29**	*p10*	31055←31393	112	P10 [Trabala vishnou gigantina nucleopolyhedrovirus]	68.1 (49/72)	*ac137*
**30**	*p26*	31468←32283	271	P26 [Agrotis ipsilon multiple nucleopolyhedrovirus]	48.2 (119/247)	*ac136*
**31**		32451→32729	92	asb138 [Agrotis segetum nucleopolyhedrovirus B]	46.7 (43/92)	*ac29*
**32**	*39k/pp31*	32785←33654	289	9K [Peridroma alphabaculovirus]	44.0 (128/291)	*ac36*
**33**	*lef-11*	33590←33997	135	LEF-11 [Chrysodeixis includens nucleopolyhedrovirus]	54.8 (57/104)	*ac37*
**34**	*bv-e31; adprase*	33919←34617	232	ADP-ribose pyrophosphatase [Spodoptera exempta nucleopolyhedrovirus]	71.8 (135/188)	*ac38*
**35**	*odv-e66*	34733→36814	693	ODV-e66 [Orgyia pseudotsugata single capsid nuclopolyhedrovirus]	45.5 (291/640)	*ac46*
**36**		36833→37321	162			
**37**	*lef-12*	37323→37973	216	Late expression factor 12 [Trabala vishnou gigantina nucleopolyhedrovirus]	37.1 (82/221)	*ac41*
**38**		37982→38212	76	hypothetical protein ManeNPV_00036 [Malacosoma neustria nucleopolyhedrovirus]	39.7 (29/73)	*ac43*
**39**	*lef-8*	38245←40875	876	lef-8 [Cryptophlebia peltastica nucleopolyhedrovirus]	71.3 (627/880)	*ac50*
**40**	*bjdp*	40881→41777	298	BJDP [Chrysodeixis includens nucleopolyhedrovirus]	30.3 (46/152)	*ac51*
**41**	*iap-3*	41754←42245	163	inhibitor of apotosis 3 [Neophasia sp. alphabaculovirus]	46.6% (76/163)	

ORF	Name	Position	aa	Top BLASTx match	% identity (range of alignment)	Notes/AcMNPV homologs
**42**	*ac52-like*	42322←42960	212	ac52 [Erannis ankeraria nucleopolyhedrovirus]	37.4 (68/182)	*ac52*
**43**	*U-box/RING-like domain*	43019→43432	137	asb115 [Agrotis segetum nucleopolyhedrovirus B]	56.6 (77/136)	*ac53*
**44**		43423←44322	299	orf30 [Euproctis digramma nucleopolyhedrovirus]	33.7 (83/246)	
**45**		44330←44548	72	hypothetical protein McnBVgp133 [Mamestra configurata nucleopolyhedrovirus B]	45.7 (32/70)	
**46**	*lef-10*	44520→44747	75	LEF-10 [Chrysodeixis chalcites nucleopolyhedrovirus]	63.6 (21/33)	*ac53a*
**47**	*vp1054*	44620→45630	336	VP1054 [Spodoptera litura nucleopolyhedrovirus II]	55.6 (189/340)	*ac54*
**48**	*ac55-like*	45691→46008	105	hypothetical protein [Chrysodeixis includens nucleopolyhedrovirus]	43.2 (35/81)	*ac55*
**49**	*ac56-like*	45950→46222	90	maco-A 131 [Mamestra configurata nucleopolyhedrovirus A]	42.6 (29/68)	*ac56*
**50**	*ac57-like*	46497→46991	164	ac57 [Malacosoma neustria nucleopolyhedrovirus]	46.3 (63/136)	*ac57*
**51**	*chaB1*	47005←47487	160	ORF-109 [Agrotis segetum nucleopolyhedrovirus A]	62.1 (54/87)	*ac58/59*
	hr3	47615 - 47798				3 repeats
**52**	*chaB2*	47870←48154	94	ChaB-like [Mythimna unipuncta nucleopolyhedrovirus]	50.9 (28/55)	*ac60*
**53**	*fp25k*	48293→48985	230	ORF98 [Spodoptera exigua multiple nucleopolyhedrovirus]	76.0 (139/183)	*ac61*
**54**	*lef-9*	49059→50564	501	lef9 [Lambdina fiscellaria nucleopolyhedrovirus]	76.3 (374/490)	*ac62*
**55**	*p13*	50584→51396	270	P13 [Spodoptera litura nucleopolyhedrovirus II]	63.8 (171/268)	
**56**	*pif-9*	51434←51700	88	se49 [Spodoptera exigua multiple nucleopolyhedrovirus]	46.4 (39/84)	*ac108*
**57**	*odv-ec43*	51718←52791	357	odv-ec43 [Cryptophlebia peltastica nucleopolyhedrovirus]	66.5 (236/358)	*ac109*
**58**	*pif-7*	52794←52958	54	PIF-7 [Mythimna unipuncta nucleopolyhedrovirus]	59.3 (32/54)	*ac110*
**59**	*vp80*	52955→54472	505	P87/VP80 [Clanis bilineata nucleopolyhedrovirus]	39.1 (66/169)	*ac104*
**60**	*p45/p48*	54492→55619	375	p12 [Apocheima cinerarium nucleopolyhedrovirus]	54.1 (211/390)	*ac103 (The ApciNPV ORF is evidently misnamed.)*
**61**	*p12*	55612←55971	119	p12 [Orgyia leucostigma nucleopolyhedrovirus]	42.0 (37/88)	*ac102*

ORF	Name	Position	aa	Top BLASTx match	% identity (range of alignment)	Notes/AcMNPV homologs
**62**	*p40; bv/odv-c42*	56036→57163	375	P40 [Agrotis ipsilon multiple nucleopolyhedrovirus]	47.3 (186/393)	*ac101*
**63**	*p6.9*	57208→57486	92			*ac100* (Identification based on conserved ORF position and Arg/Ser-rich nature of this short polypeptide)
**64**	*lef-5*	57480→58301	273	late expression factor 5 [Ectropis obliqua nucleopolyhedrovirus]	63.1 (169/268)	*ac99*
**65**	*38k*	58194→59111	305	38k [Hyposidra talaca nucleopolyhedrovirus]	60.2 (183/304)	*ac98*
**66**	*pif-4*	59160←59678	172	per os infectivity factor 4 [Orgyia pseudotsugata single capsid nuclopolyhedrovirus]	61.4 (105/171)	*ac96*
**67**	*dnahel*	59635→63285	1216	helicase/P143 [Clanis bilineata nucleopolyhedrovirus]	52.1 (655/1257)	*ac95*
**68**	*odv-e25*	63376←64053	225	ODV-E25 [Clanis bilineata nucleopolyhedrovirus]	73.2 (164/224)	*ac94*
**69**	*p18*	64081←64578	165	hypothetical protein [Pseudoplusia includens SNPV IE]	69.9% (109/156)	*ac93*
**70**	*p33*	64577→65347	256	P33 [Trabala vishnou gigantina nucleopolyhedrovirus]	64.7 (163/252)	*ac92*
	hr4	65587 - 66090				7 repeats
**71**	*lef-4*	66218←67618	466	lef-4 [Peridroma alphabaculovirus]	53.7 (253/471)	*ac90*
**72**	*vp39*	67617→68684	355	VP39 [Urbanus proteus nucleopolyhedrovirus]	56.4% (171/303)	*ac89*
**73**	*cg30*	68772→69719	315	CG30 [Perigonia lusca single nucleopolyhedrovirus]	28.7 58/202)	*ac88*
**74**	*vp91*	69796←72333	845	VP91 CAPSID [Agrotis segetum nucleopolyhedrovirus A]	48.9 (406/831)	*ac83*
**75**	*tlp20*	72302→72946	214	agip95 [Agrotis ipsilon multiple nucleopolyhedrovirus]	67.6 (71/105)	*ac82*
**76**	*ac81-like*	72750→73481	243	hypothetical protein [Rachiplusia nu nucleopolyhedrovirus]	69.7 (131/188)	*ac81*
**77**	*gp41*	73468→74484	338	GP41 [Clanis bilineata nucleopolyhedrovirus]	72.3% (214/296)	*ac80*
**78**	*ac78-like*	74484→74816	110	maco-A 105 [Mamestra configurata nucleopolyhedrovirus A]	37.2 (32/86)	*ac78*
**79**	*vlf-1*	74857→75990	377	VLF-1 [Chrysodeixis includens nucleopolyhedrovirus]	83.1 (285/343)	*ac77*
**80**	*vef*	76003→78273	756	enhancin-like [Choristoneura fumiferana multiple nucleopolyhedrovirus]	38.1 (279/732)	

ORF	Name	Position	aa	Top BLASTx match	% identity (range of alignment)	Notes/AcMNPV homologs
**81**	*ac76-like*	78289→78546	85	hypothetical protein [Orgyia leucostigma nucleopolyhedrovirus]	82.4 (70/85)	*ac76*
**82**	*ac75-like*	78557→78943	128	hypothetical protein [Pseudoplusia includens SNPV IE]	51.9 (67/129)	*ac75*
**83**	*dnapol*	78991←82122	1043	DNA polymerase [Peridroma alphabaculovirus]	55.2 (568/1029)	*ac65*
**84**	*desmop*	81983→84367	794	desmoplakin [Rachiplusia nu nucleopolyhedrovirus]	62.0% (62/100)	*ac66*
**85**	*lef-3*	84436←85713	425	lef3 [Peridroma alphabaculovirus]	40.7 (133/327)	*ac67*
**86**	*pif-6*	85715→86131	138	22.3kDa/pif-6 [Trichoplusia ni single nucleopolyhedrovirus]	56.2 (73/130)	*ac68*
**87**	*mtase*	86073→86891	272	se78 [Spodoptera exigua multiple nucleopolyhedrovirus]	56.4 (149/264)	*ac69*
**88**	*iap-2*	86875→87729	284	IAP-2 [Chrysodeixis chalcites nucleopolyhedrovirus]	37.3 (112/300)	*ac71*
**89**	*p26b*	87795→88487	230	P26b [Chrysodeixis includens nucleopolyhedrovirus]	42.2% (100/237)	
**90**	*p35/p49*	88536←89645	369	P35/P49-like [Choristoneura fumiferana granulovirus]	34.2 (129/377)	*ac135*
	hr5	89806 - 89989				3 repeats
**91**	*orf603*	90140→90784	214	Clas51 [Clostera anastomosis granulovirus B]	45.5 (102/224)	*ac7*
	hr6	90852 - 91175				5 repeats
**92**	*pep*	91218→92213	331	calyx/pep [Cryptophlebia peltastica nucleopolyhedrovirus]	48.9 (155/317)	*ac131*
**93**	*rr2*	92251←93267	338	rr2 [Hemileuca sp. nucleopolyhedrovirus]	73.5 (247/336)	
**94**	*ac19-like*	93327←93665	112	ac19-like protein [Cryptophlebia peltastica nucleopolyhedrovirus]	42.9 (30/70)	*ac19*
**95**	*ac18-like*	93658→94818	386	ORF-45 [Agrotis segetum nucleopolyhedrovirus A]	40.1 (156/389)	*ac18*
	hr7	94892 - 95075				3 repeats
**96**	*bro-a*	95210←96211	333	baculovirus repeated ORF [Anticarsia gemmatalis multiple nucleopolyhedrovirus]	74.7 (254/320)	
**97**	*pif-1*	96286←97905	539	per os infectivity factor 1 [Apocheima cinerarium nucleopolyhedrovirus]	62.9% (313/498)	*ac119*
**98**		97990←98316	108	hypothetical protein EupsNPV_gp105 [Euproctis pseudoconspersa nucleopolyhedrovirus]	37.7% (26/69)	*ac117*
**99**		98365←98700	111	se39 [Spodoptera exigua multiple nucleopolyhedrovirus]	51.4 (55/107)	
**100**	*sod*	98718←99173	151	sod [Peridroma alphabaculovirus]	74.8 (113/151)	*ac31*
**101**		99237→99662	141			

ORF	Name	Position	aa	Top BLASTx match	% identity (range of alignment)	Notes/AcMNPV homologs
**102**	*ac84-like*	99665→100096	143			HHpred match at 96.17% for “Uncharacterized 21.7 kDa protein in GP41-PNK intergenic region”. (eg. Ac84)
**103**	*lef-2*	100125←100778	217	lef2 primase associated protein [Peridroma alphabaculovirus]	49.0 (101/206)	*ac6*
**104**		100720←100992	90	hypothetical protein KM622_gp012 [Spodoptera exempta nucleopolyhedrovirus]	66.7 (22/33)	
**105**	*p24*	101091→101759	222	p24 [Hyposidra talaca nucleopolyhedrovirus]	60.8 (132/217)	*ac129*
**106**	*gp16*	101766→102080	104	GP16 [Buzura suppressaria nucleopolyhedrovirus]	48.5 (47/97)	*ac130*
**107**	*pkip*	102104→102625	173	PKIP [Clanis bilineata nucleopolyhedrovirus]	43.9 (82/187)	*ac24*
**108**		102642→103100	152			
**109**	*arif-1*	103172←103957	261	arif-1 [Peridroma alphabaculovirus]	31.9 (73/229)	*ac20/21*
**110**	*pif-2*	103996→105147	383	pif-2 [Erannis ankeraria nucleopolyhedrovirus]	70.7 (270/382)	*ac22*
**111**	*p47*	105162→106376	404	p47 [Hemileuca sp. nucleopolyhedrovirus]	63.5 (256/403)	*ac40*
**112**		106492→107520	342	hypothetical protein [Parapoynx stagnalis nucleopolyhedrovirus]	51.0 (175/343)	*ac11*
	hr8	107631 - 107955				5 repeats
**113**	*exon0*	108029→108790	253	[Agrotis segetum nucleopolyhedrovirus B]	49.1 (112/228)	*ac141*
**114**	*p49*	108823→110277	484	P49 [Clanis bilineata nucleopolyhedrovirus]	62.8 (289/460)	*ac142*
**115**	*odv-e18*	110255→110509	84	occlusion-derived virus envelope protein 18 [Pseudoplusia includens SNPV IE]	72.6 (61/84)	*ac143*
**116**	*odv-ec27*	110533→111432	299	ODV-E27 [Lymantria xylina nucleopolyhedrovirus]	59.7 (176/295)	*ac144*
**117**	*ac145-like*	111433←111711	92	ac145 [Helicoverpa armigera nucleopolyhedrovirus]	62.0 (57/92)	*ac145*
**118**	*ie-1*	111771←113720	649	IE-1 [Mythimna unipuncta nucleopolyhedrovirus]	39.5 (152/385)	*ac147*
**119**	*ac146-like*	113758→114357	199	ac146 [Malacosoma neustria nucleopolyhedrovirus]	36.7 (73/199)	*ac146*
	hr9	114471 - 114666				2 repeats
**120**	*odv-e18*	114698→114877	59	odv-e18 [Clostera anachoreta granulovirus]	89.47 (34/38)	*ac143*
**121**		114940←115479	179	hypothetical protein Leryth_021875 [Lithospermum erythrorhizon]	40.4 (23/57)	
**122**	*ac146-like*	115978←116199	73	ORF7 [Pieris brassicae granulovirus]	65.5 (38/58)	

ORF	Name	Position	aa	Top BLASTx match	% identity (range of alignment)	Notes/AcMNPV homologs
**123**		116315→116683	122	cyun52 [Cyclophragma undans nucleopolyhedrovirus]	31.8 (40/126)	
**124**	*pif-3*	116753→117391	212	pif-3 [Agrotis segetum nucleopolyhedrovirus B]	59.5 (113/190)	*ac115*
**125**	*parg*	117407→118789	460	asb055 [Agrotis segetum nucleopolyhedrovirus B]	30.9 (100/324)	
**126**	*ac106/107 -like*	118833→119486	217	AC106 [Trabala vishnou gigantina nucleopolyhedrovirus]	64.7 (152/235)	*ac106/107*
**127**	*dut*	119550→119978	142	dutpase [Peridroma alphabaculovirus]	47.9 (68/142)	
**128**	*nrk-1*	120131→121171	346	Nrk1 [Cryptophlebia peltastica nucleopolyhedrovirus]	44.0 (118/268)	
	hr10	121311 - 121710				5 repeats
