# Supplementary material for: The Genome Sequences of Baculoviruses from the Tufted Apple Bud Moth, Platynota idaeusalis, Reveal Recombination Between an Alphabaculovirus and a Betabaculovirus from the Same Host
Source: Viruses. 2025 Jan 30;17(2):202. doi: 10.3390/v17020202 (PMC11861948; doi:10.3390/v17020202)
Supplement: Supplementary file 1 [file viruses-17-00202-s001.zip › Table S2.pdf]

Table S2. PlidGV-2683 open reading frames (ORFs) and direct repeat regions (*dr*s)

ORF	Name	Position	aa	Top BLASTx match	% identity (range of alignment)	Notes/AcMNPV homologues
**1**	*gran*	1→747	248	granulin [Erinnyis ello granulovirus]	97.6% (243/248)	*ac8*
**2**	*p78/83*	744←1097	117	Maph2 [Matsumuraeses phaseoli granulovirus]	50.4% (57/113)	*ac9*
**3**	*pk-1*	1078→1902	274	PK-1 [Betabaculovirus arrapae]	70.1% (195/278)	*ac10*
**4**		1922←2500	192	ORF4 [Pieris rapae granulovirus]	55.7% (107/192)	
**5**		2490→2738	82	unknown [Pieris rapae granulovirus]	62.3% (48/77)	
	dr1	2764 - 2993				6 27-bp repeats, in two clusters of three tandem direct repeats
**6**	*ie-1*	3103←4491	462	ie-1 [Betabaculovirus arrapae]	53.4% (214/401)	*ac147*
**7**	*ac146-like*	4530→5087	185	hypothetical protein [Pieris rapae granulovirus]	48.9% (90/184)	*ac146*
**8**	*chtb1*	5116←5421	101	ORF8 [Pieris rapae granulovirus]	71.3% (72/101)	*ac145*
**9**		5428→5964	178	hypothetical protein Leryth_021875 [Lithospermum erythrorhizon]	38.6% (22/57)	
**10**	*odv-e18*	6025←6303	92	odv-e18 [Cnaphalocrocis medinalis granulovirus]	77.1% (74/96)	*ac143*
**11**	*p49*	6304←7680	458	p49 [Pieris rapae granulovirus]	63.0% J(289/459)	*ac142*
**12**	*odv-e56*	7867←8928	353	ODV-E56 [Choristoneura fumiferana granulovirus]	77.2% (267/346)	*ac148*
**13**	*ac29-like*	9330→9509	59			HHpred: probability 97.49% match to DUF919 (AcMNPV-C6 ORF ac29)
**14**	*pep-1*	9510←10016	168	polyhedron envelope protein 1 [Darna trima granulovirus]	75.7% (131/173)	*ac131*
**15**	*pep-p10*	10091→11068	325	polyhedron envelope/P10 fusion protein [Erinnyis ello granulovirus]	68.0% (232/341)	
**16**	*pep-2*	11084→11497	137	pep-2 [Matsumuraeses phaseoli granulovirus]	65.9% (89/135)	*ac131*
**17**		11791←12639	282	hypothetical protein BD410DRAFT_838475 [Rickenella mellea]	25.7% (39/152)	
	dr2	12785 - 12928				Six tandem 24-bp direct repeats
**18**		13005←14015	336	protein TsetseEP-like [Cherax quadricarinatus]	36.6% (26/71)	
**19**		14039←14866	275	hypothetical protein [Cryptophlebia leucotreta granulovirus]	35.1% (72/205)	
	dr3	15552 - 15875				12 21-bp direct repeats (mostly tandem, some dispersed)

**20**		16488→17453	321	hypothetical protein D1Q00_gp021 [Trichoplusia ni granulovirus LBIV-12]	40.2% (41/102)	
**21**		17812→17979	55			
**22**		18396→19604	402	PrGVORF24 [Betabaculovirus arrapae]	31.7% (135/426)	
**23**	*F protein*	19755→21470	571	hypothetical protein PPFHPHBJ_00005 [Cydia pomonella granulovirus]	57.1% (336/588)	*ac23*
**24**		21527→22540	337	ORF27 [Pieris rapae granulovirus]	46.2% (36/78)	
**25**		22543←23214	223	Maph29 [Matsumuraeses phaseoli granulovirus]	37.1% (96/259)	
**26**		23220←23792	190	unknown [Pieris rapae granulovirus]	59.2% (113/191)	
**27**	*pif-3*	23818→24381	187	PrGVORF30 [Betabaculovirus arrapae]	54.6% (100/183)	*ac115*
**28**	*odv-e66*	24405←26717	770	odv-e66 [Darna trima granulovirus]	64.2% (497/774)	*ac46*
**29**		26754→27068	104	ORF39 similar to XcGV ORF34 [Cydia pomonella granulovirus]	71.8% (74/103)	
**30**		27087←27341	84			
**31**		27457→27837	126	agip26 [Agrotis ipsilon multiple nucleopolyhedrovirus]	37.1% (53/143)	
**32**	*lef-2*	27839→28360	173	LEF-2 [Choristoneura fumiferana granulovirus]	55.4% (93/168)	*ac6*
**33**		28341→28586	81	ORF42 similar to XcGV ORF36 [Cydia pomonella granulovirus]	46.3% (38/82)	
**34**		28591←28914	107	PrGVORF35 [Betabaculovirus arrapae]	36.8% (42/114)	
**35**		28907←29260	117			
**36**	*metalloproteinase*	29313←30692	459	mp-nase [Pieris rapae granulovirus]	52.2% (210/402)	
**37**	*p13*	30650→31465	271	p13 [Cryptophlebia leucotreta granulovirus]	66.4% (180/271)	
**38**	*pif-2*	31474→32589	371	ORF48 pif-1 [Cydia pomonella granulovirus] (This is actually *pif-2*)	69.7% (260/373)	*ac22*
**39**		32586←32774	62	Maph42 [Matsumuraeses phaseoli granulovirus]	47.6% (30/63)	
**40**		32789→36016	1075	ORF42 [Pieris rapae granulovirus]	63.8% (95/149)	
**41**	*ac106/107*	36044←36658	204	unknown [Choristoneura fumiferana granulovirus]	73.3% (151/206)	*ac106/107*
**42**	*pif-7*	36664→36816	50	hypothetical protein [Diatraea saccharalis granulovirus]	62.2% (28/45)	*ac110*
**43**		36873→37247	124	hypothetical protein ClanGV_gp042 [Clostera anachoreta granulovirus]	31.7% (38/120)	
**44**	*ubiquitin-like*	37273←37557	94	ubiquitin-like protein [Diatraea saccharalis granulovirus]	85.1% (80/94)	*ac35*
**45**	*odv-ec43*	37641→38678	345	unknown [Pieris rapae granulovirus]	66.2% (235/355)	*ac109*
**46**		38682→38840	52	ORF56 similar to XcGV ORF54 [Cydia pomonella granulovirus]	55.1% (27/49)	*ac108*

**47**		38790←39170	126	Maph49 [Matsumuraeses phaseoli granulovirus]	40.4% (44/109)	
**48**	*pp31/39k*	39179←40009	276	39K [Pieris rapae granulovirus]	57.3% (154/269)	*ac36*
**49**	*lef-11*	39993←40289	98	LEF-11 [Plodia interpunctella granulovirus]	60.7% (54/89)	*ac37*
**50**	*sod*	40316←40786	156	SOD [Plodia interpunctella granulovirus]	67.5% (106/157)	*ac31*
**51**	*p10*	41219←41437	72	P10 [Choristoneura fumiferana granulovirus]	69.8% (44/63)	*ac137*
**52**	*p74*	41469←43442	657	ORF60 p74 [Cydia pomonella granulovirus]	64.9% (449/692)	*ac138*
**53**		43491→44084	197	ORF109 [Cydia pomonella granulovirus]	32.4% (57/176)	
**54**	*acetyltransferase-like (actr)*	44664←45278	204	unknown [Pieris rapae granulovirus]	66.2% (131/198)	
	dr4	45404 - 45711				11 28-bp direct tandem repeats
**55**		45970←46263	97	hypothetical protein [Cryptophlebia leucotreta granulovirus]	69.8% (44/63)	
**56**	*p47*	46323→47498	391	P47 [Pieris rapae granulovirus]	65.3% (256/392)	*ac40*
**57**	*adprase*	47542→48210	222	bv-e31 [Matsumuraeses phaseoli granulovirus]	77.5% (172/222)	*ac38*
**58**		48207←48761	184	ORF70 [Cydia pomonella granulovirus]	38.0% (63/166)	
**59**	*p24*	48779→49318	179	P24 capsid protein [Choristoneura fumiferana granulovirus]	63.1% (106/168)	*ac129*
**60**		49315→49587	90	unknown [Choristoneura fumiferana granulovirus]	45.5% (40/88)	
**61**	*lef-1*	49588←50286	232	lef-1 [Clostera anastomosis granulovirus Henan]	61.2% (142/232)	*ac14*
**62**	*pif-1*	50302→51912	536	PIF-1 [Choristoneura fumiferana granulovirus]	67.8% (362/534)	*ac119*
**63**	*fgf-1*	51964←52629	221	FGF-1 [Betabaculovirus arrapae]	53.0% (116/219)	*ac32*
**64**		52674←52979	101	hypothetical protein [Pieris rapae granulovirus]	47.1% (48/102)	
**65**	*chtb2*	53146→53607	153	hypothetical protein [Cryptophlebia leucotreta granulovirus]	44.8% (69/154)	*ac150*
**66**	*lef-6*	53604←53858	84	LEF-6 [Betabaculovirus arrapae]	50.8% (33/65)	*ac28*
**67**	*dbp*	53879←54727	282	DBP [Pieris rapae granulovirus]	67.6% (190/281)	*ac25*
**68**		54672←54926	84	Maph72 [Matsumuraeses phaseoli granulovirus]	54.3% (38/70)	
**69**		54862←55479	205	hypothetical protein [Pieris brassicae granulovirus]	40.7% (77/189)	
**70**	*p45/p48*	55457→56623	388	P45/P48 [Betabaculovirus arrapae]	75.7% (292/386)	*ac103*
**71**	*p12-like*	56648→56983	111	ORF84 similar to AcMNPV ORF102 [Cydia pomonella granulovirus]	57.6% (57/99)	*ac102*
**72**	*p40; bv/odv-c42*	57046→58182	378	PrGVORF70 [Betabaculovirus arrapae]	70.0% (262/377)	*ac101*
**73**	*p6.9*	58213→58371	52			*ac100*; Identified by its conserved position and high Arg and Ser content of encoded peptide

**74**	*lef-5*	58401←59138	245	LEF-5 [Betabaculovirus arrapae]	66.9% (166/248)	*ac99*
**75**	*38k*	59085→60008	307	38K [Diatraea saccharalis granulovirus]	67.4% (196/291)	*ac98*
**76**		59983→60270	95	unnamed protein product [Diatraea saccharalis]	42.3% (40/94)	
**77**	*odv-e28/pif-4*	60349←60828	159	hypothetical protein [Cryptophlebia leucotreta granulovirus]	56.6% (90/159)	*ac96*
**78**	*dnahel*	60812→64201	1129	helicase-1 [Betabaculovirus arrapae]	64.5% (734/1138)	*ac95*
**79**	*odv-e25*	64207←64848	213	ORF91 ODV-E25 [Cydia pomonella granulovirus]	84.0% (179/213)	*ac94*
**80**	*p18*	64897←65376	159	p18 [Darna trima granulovirus]	54.8% (86/157)	*ac93*
**81**	*p33*	65389→66144	251	p33 [Pieris rapae granulovirus]	66.1% (166/251)	*ac92*
**82**	*iap-6*	66146←66805	219	iap-6 [Matsumuraeses phaseoli granulovirus]	40.5% (96/237)	
**83**	*lef-4*	66807←68144	445	LEF-4 [Pieris rapae granulovirus]	56.9% (352/476)	*ac90*
**84**	*vp39*	68207→69088	293	VP39 capsid [Betabaculovirus arrapae]	66.6% (195/293)	*ac89*
**85**	*odv-ec27*	69132→69992	286	ODV-e27 [Betabaculovirus arrapae]	72.7% (208/286)	*ac144*
**86**		70552←71658	368	PrGVORF83 [Betabaculovirus arrapae]	46.9% (176/375)	
**87**		71734→72000	88	Maph90 [Matsumuraeses phaseoli granulovirus]	45.0% (45/100)	
**88**	*vp91*	71979←74069	696	ORF101 vp91 capsid [Cydia pomonella granulovirus]	42.4% (296/698)	*ac83*
**89**	*tlp*	74038→74433	131	TLP-20 [Plodia interpunctella granulovirus]	35.2% (45/128)	*ac82*
**90**	*ac81*	74387→75034	215	Maph93 [Matsumuraeses phaseoli granulovirus]	70.5% (155/220)	*ac81*
**91**	*gp41*	74979→75842	287	gp41 [Cydia pomonella granulovirus]	72.0% (206/286)	*ac80*
**92**		75968→76708	246	baculoviral IAP repeat-containing protein 3 isoform X2 [Nymphalis io]	49.5% (143/289)	All matches returned by BLAST were from lepidopterans
**93**	*ac78*	76718→76996	92	PrGVORF89 [Betabaculovirus arrapae]	41.8% (43/103)	*ac78*
**94**	*vlf-1*	76932→78074	380	VLF-1 [Betabaculovirus arrapae]	74.9% (271/362)	*ac77*
**95**	*ac76*	78081→78335	84	hypothetical protein [Psilogramma increta granulovirus]	79.8% (67/84)	*ac76*
**96**	*ac75*	78393→78848	151	unknown [Choristoneura fumiferana granulovirus]	68.5% (102/149)	*ac75*
**97**		78838←79224	128	hypothetical protein [Diatraea saccharalis granulovirus]	32.8% (41/125)	
**98**	*dnapol*	79293←82406	1037	DNA polymerase [Pieris rapae granulovirus]	68.5% (718/1049)	*ac65*
**99**	*desmop*	82342→84402	686	desmoplakin [Pieris rapae granulovirus]	37.7% (292/775)	*ac66*
	dr5	84515 - 84682				6 28-bp tandem direct repeats
**100**	*lef-3*	84881←85912	343	lef-3 [Clostera anastomosis granulovirus B]	50.4% (174/345)	*ac67*
**101**	*pif-6*	85878→86261	127	unknown [Choristoneura fumiferana granulovirus]	70.7% (87/123)	*ac68*
**102**		86299→86802	167	hypothetical protein [Pieris rapae granulovirus]	52.8% (86/163)	
**103**	*iap-5*	86820→87632	270	inhibitor of apoptosis 5 [Phthorimaea operculella granulovirus]	58.1% (155/267)	

**104**	*lef-9*	87613→89118	501	lef-9 [Matsumuraeses phaseoli granulovirus]	74.3% (362/487)	*ac62*
**105**	*fp25k*	89105→89563	152	FP [Betabaculovirus arrapae]	73.5% (111/151)	*ac61*
**106**	*DNA ligase*	89560←91245	561	DNA ligase [Betabaculovirus arrapae]	65.0% (368/566)	
**107**		91363→91584	73	Clas108 [Clostera anastomosis granulovirus B]	49.3% (35/71)	
**108**	*fgf-2*	91590←92771	393	FGF-2 [Betabaculovirus arrapae]	51.6% (183/355)	*ac32*
**109**		92881→93183	100	PrGVORF106 [Betabaculovirus arrapae]	64.6% (62/96)	
**110**		93221←93871	216			
**111**	*alk-exo*	93939→95141	400	ALK-EXO [Betabaculovirus arrapae]	63.2% (251/397)	*ac133*
**112**	*helicase-2*	95065→96363	432	helicase-2 [Betabaculovirus arrapae]	58.0% (258/445)	
**113**		96336←97298	320	ORF109 [Pieris rapae granulovirus]	40.4% (95/235)	
**114**	*lef-8*	97359←99932	857	LEF-8 [Betabaculovirus arrapae]	71.7% (631/880)	*ac50*
**115**		99977→100354	125	Maph118 [Matsumuraeses phaseoli granulovirus]	36.3% (37/102)	
**116**		100371←100544	57	ORF112 [Pieris rapae granulovirus]	58.6% (34/58)	
**117**	*ac53*	100543→100950	135	Maph120 [Matsumuraeses phaseoli granulovirus]	72.9% (97/133)	*ac53*
**118**		100940←101842	300	Clas118 [Clostera anastomosis granulovirus B]	44.1% (94/213)	
**119**		101851←102132	93	unknown [Choristoneura fumiferana granulovirus]	56.4% (31/55)	
**120**	*lef-10*	102017→102250	77	lef-10 [Pieris rapae granulovirus]	60.0% (42/70)	*ac53a*
**121**	*vp1054*	102135→103118	327	VP1054 [Betabaculovirus arrapae]	62.0% (204/329)	*ac54*
**122**		103158→103346	62			
**123**	*fgf-3*	103429→104214	261	FGF-3 [Betabaculovirus arrapae]	52.9% (139/263)	*ac32*
**124**	*egt*	104217←105545	442	egt [Betabaculovirus arrapae]	53.3% (242/454)	*ac15*
**125**	*me53*	105644→106588	314	ME-53 [Betabaculovirus arrapae]	58.3% (179/307)	*ac139*
